# Art therapy for depression: a systematic review and meta-analysis

**DOI:** 10.3389/fpsyt.2026.1789633

**Published:** 2026-07-23

**Authors:** Ronja Joschko, Stephanie Roll, Weronika A. Grabowska, Stefan N. Willich, Anne Berghöfer

**Affiliations:** 1 Institute of Social Medicine, Epidemiology and Health Economics, Charité – University Medical Center Berlin, corporate member of Freie Universität Berlin and Humboldt-Universität zu Berlin, Berlin, Germany; 2 Institute for Biostatistics and Informatics in Medicine and Aging Research, Universität Rostock, Rostock, Germany

**Keywords:** art therapy, creative therapy, depression, meta-analysis, systematic review, therapy effectiveness evaluation, visual art therapy

## Abstract

**Introduction:**

Depression is among the leading causes of disability globally. Therefore, exploring the various non-medical treatment options for this condition is particularly important. The aim of the review was to assess the effect of art therapy on depressive symptoms.

**Methods:**

The foundation of this review is a pre-planned, explorative, secondary analysis of a previously published umbrella review, encompassing the databases Cochrane Library, Embase, MEDLINE, CINAHL, ERIC, American Psychological Association PsycArticles, American Psychological Association PsycInfo, PSYNDEX, the German Clinical Trials Register, and ClinicalTrials.gov. Included were all randomized trials with any patient population receiving active visual art therapy. The outcome was depressive symptoms measured by depression assessment instruments. We followed the Preferred Reporting Items for Systematic Reviews and Meta-Analyses (PRISMA) guidelines and conducted a bias assessment using a modified Cochrane risk of bias tool. Data was pooled using a random-effects model and visualized in forest plots. A pooled standardized mean difference (SMD) with hedges g was calculated to measure the reduction of depressive symptoms.

**Results:**

Of 3,100 identified reports we included 26 studies. Of these, 19 studies with 997 patients were eligible for inclusion in the meta-analysis. Overall, we found a standardized mean difference of 0.53 (95% CI: 0.30 to 0.76) for depressive symptoms, favoring the intervention group. Main sources of variation were different types of control groups, methodological quality, and patient populations.

**Conclusion:**

Our results suggest that art therapy is associated with improved depressive symptoms. Therefore, art therapy should be accessible as complementary treatment for patients suffering from depressive symptoms.

## 1 Introduction

Mental health disorders are amongst the largest burden of diseases globally, with depression being the most common one (1). Depression can profoundly impact the life of individuals experiencing it, and it is estimated that around one in three people will experience a major depressive episode during their lifetime (2). The costs associated with the two most common mental disorders, anxiety and depression, were estimated to be around US$ 1 trillion globally in 2020, a number expected to increase to US$ 6 trillion by the year 2030 (3).The standard treatment of unipolar depression in adults, according to medical guidelines, includes the use of psychotherapy and pharmacotherapy (4). However, mental health disorders, such as depression, can be difficult to treat, and acute facilities and rehab facilities often offer a wide array of non-pharmacologically and complementary treatment options, like movement therapies, light therapy, and art therapy (5, 6).

The umbrella term ‘art therapy’ refers to therapeutic approaches that integrate artistic elements, such as music, dance, theatre or literature, into treatment. Art therapy is used for a wide range of treatment indications and may pursue curative, rehabilitative, preventive, palliative or corrective treatment goals (7–11), they are most widely established in the treatment of mental disorders, whereby the underlying theoretical concept may be rooted in psychodynamic or analytical, cognitive or behaviour-oriented psychotherapy (12, 13). In the art therapy process, the focus is not primarily on the artistic product, but on the individual expression of inner worlds of experience through visual means (14).

The exact mechanism through which art therapy might alleviate symptoms in patients is not yet fully established and remains a source of investigation. A non-verbal approach appears to open up alternative ways of addressing psychological conflicts, particularly in situations where language is limited or cognitive processing seems difficult (15, 16). Generally effective elements of art therapy include the relationship between therapist and patient, mindfulness, and positive group effects, such as a sense of belonging and connectedness (17, 18). Specific mechanisms of action include the safe space in which self-exploration can be approached in a playful manner (19), the non-verbal approach to and engagement with one’s own feelings and emotions (20), and the strengthening of self-efficacy (21). Although art therapy is commonly used for a wide range of patients and conditions, the empirical evidence for its effectiveness appears to be heterogeneous (22). A comprehensive scoping review by the World Health Organisation (WHO) identified and compiled numerous studies on artistic therapies (23). The analysis of over 900 studies demonstrated the potential of artistic therapies to promote health, prevent illness and support people in coping with illness. However, the review lacked an assessment of the quality of the included studies and a systematic literature search. A recent systematic review examined the evidence for art therapy in patients with anxiety disorders and/or depression. The authors considered only English-language publications and did not carry out a meta-analysis (22).

Therefore, we conducted an extensive systematic review, encompassing all randomized controlled trials (RCTs) examining the effectiveness of art therapy regarding any patient health outcomes. The analysis of the overall results has been published separately (24), while the present report will exclusively focus on the use of art therapy for depressive symptoms. Furthermore, we were interested in how different factors, such as presence of a therapeutical element, the type of control group, depression assessment, analysis type, age of the patients and treatment indication, might influence the effect of the intervention.

## 2 Methods

### 2.1 Stakeholder involvement

To inform the scope of the review, the list of keywords to be compiled for the literature search, and the planning of the subgroup analyses stakeholders were involved prior to the start of the systematic review. The stakeholders comprised five individuals actively working in art therapy (one doctor, one psychologist, three art therapists) and 12 patients, who were interviewed regarding the indications, therapeutic goals and therapeutic outcomes they considered important from their perspective.

### 2.2 Literature search

The protocol for this systematic review has been registered with PROSPERO (CRD42021233272) and specifics of the methods of the systematic literature search are reported in detail elsewhere (24, 25). The literature search aimed to provide a comprehensive overview without applying language or publication date restrictions. The search strategy combined three components: terms related to art, terms related to the therapeutic use of art, and an adapted randomized controlled trial (RCT) filter. The strategy was adjusted to fit the requirements of each database and was peer reviewed by two authors according to the PRESS guideline. Following Cochrane recommendations, the researchers searched the Cochrane Library, MEDLINE, and EMBASE via Ovid, as well as CINAHL, ERIC, APA PsycArticles, APA PsycInfo, and PSYNDEX via EBSCOHost. Additional searches included the German Clinical Trials Register (DRKS) and ClinicalTrials.gov. The WHO International Clinical Trials Registry Platform (ICTRP) could not be accessed at the time and was therefore excluded. A manual search of the Journal of Creative Arts Therapies was also conducted. All systematic searches, except the hand search, took place in March 2021. Data was first extracted on March 15th, 2021.

### 2.3 Study selection and data extraction

The study selection process consisted of two independent review stages using Covidence software: first, screening titles and abstracts, and second, evaluating full texts of relevant studies. References from identified reviews were also checked to identify any missing studies. Study protocols were matched with published studies for bias assessment and ongoing study tracking. Data on interventions, control groups, participants, study characteristics, outcomes, and potential sources of bias were systematically extracted using a standardized form.

### 2.4 Inclusion and exclusion criteria

For this analysis, we focused on RCTs with a patient population of any age and treatment indication, if depression was a measured outcome. We categorized patients as children (<18 years), adults (18–65 years), or older adults (>65 years). Given that most studies report the average age of participants, rather than individual ages, we categorized studies according to their participant’s average age.

Following our protocol, we included any intervention that utilized any form of active visual art therapy (AVAT) and excluded studies with intervention groups receiving a multimodal treatment package with possibly more than one effective intervention (i.e. a combined dancing and painting intervention).

### 2.5 Statistical analysis

We grouped all studies depending on whether they utilized an intention to treat (ITT), or a per protocol (PP) analysis. While ITT analyses are known to yield weaker treatment effects (26), they could have higher external validity and a more accurate representation of real world effects. The studies used two different methods to record the outcome. Some studies measured results only once, after the intervention (post-test (PT)), while others measured before and after the intervention, to take a mean difference score (change from baseline (CfB)).

A random-effects model was selected for the meta-analysis, taking into account the varying characteristics of the studies. A standardised mean difference (SMD) based on Hedges’ g was chosen as the effect size to account for the influence of small studies (27). We conducted explorative subgroup analyses, to further understand the effects of AVAT and the factors potentially influencing it.

### 2.6 Selection of control groups

The selection of appropriate control groups is of special importance when trying to assess the effectiveness of an intervention. We sorted all studies into the following control group categories, to determine their possible influence on the results: “Waitlist”, “treatment as usual (TAU)”, “no intervention” and “other”. The category “other” included activities that we suspected might have an inherent therapeutical benefit, such as psychotherapy.

### 2.7 Definition of therapeutical element

Since the definition of AVAT can vary considerably across people and institutions, we categorized the interventions according to whether they contained a therapeutical element (TE). If the intervention incorporated elements of self-reflection, exploration of feelings and emotions, or other psychotherapeutic elements, we coded the intervention as having a TE. If the intervention was purely occupational, such as coloring-in shapes, we coded the intervention as having no TE.

### 2.8 Definition of outcome

The primary analysis examined the effect of AVAT on depressive symptoms. Depressive symptoms were assessed through standardized scales and questionnaires. If more than one measure of depression was reported, we extracted the one that was most relevant to the patient population. Since not all studies used the same scale, we calculated a standardized mean difference (SMD) using Hedges’ g. The SMD was calculated by dividing the CfB or PT scores (if both were reported we used CfB) by the pooled standard deviation.

Secondary analyses involve distinguishing between different methods of measuring depression, since the observed symptoms of depression can differ from the subjective perception of the illness (28). Further secondary analyses examine the use of AVAT for various indications, distinguish between studies based on the presence of therapeutic elements and various therapeutic approaches in control groups.

## 3 Results

### 3.1 Literature search

**Figure 1:**
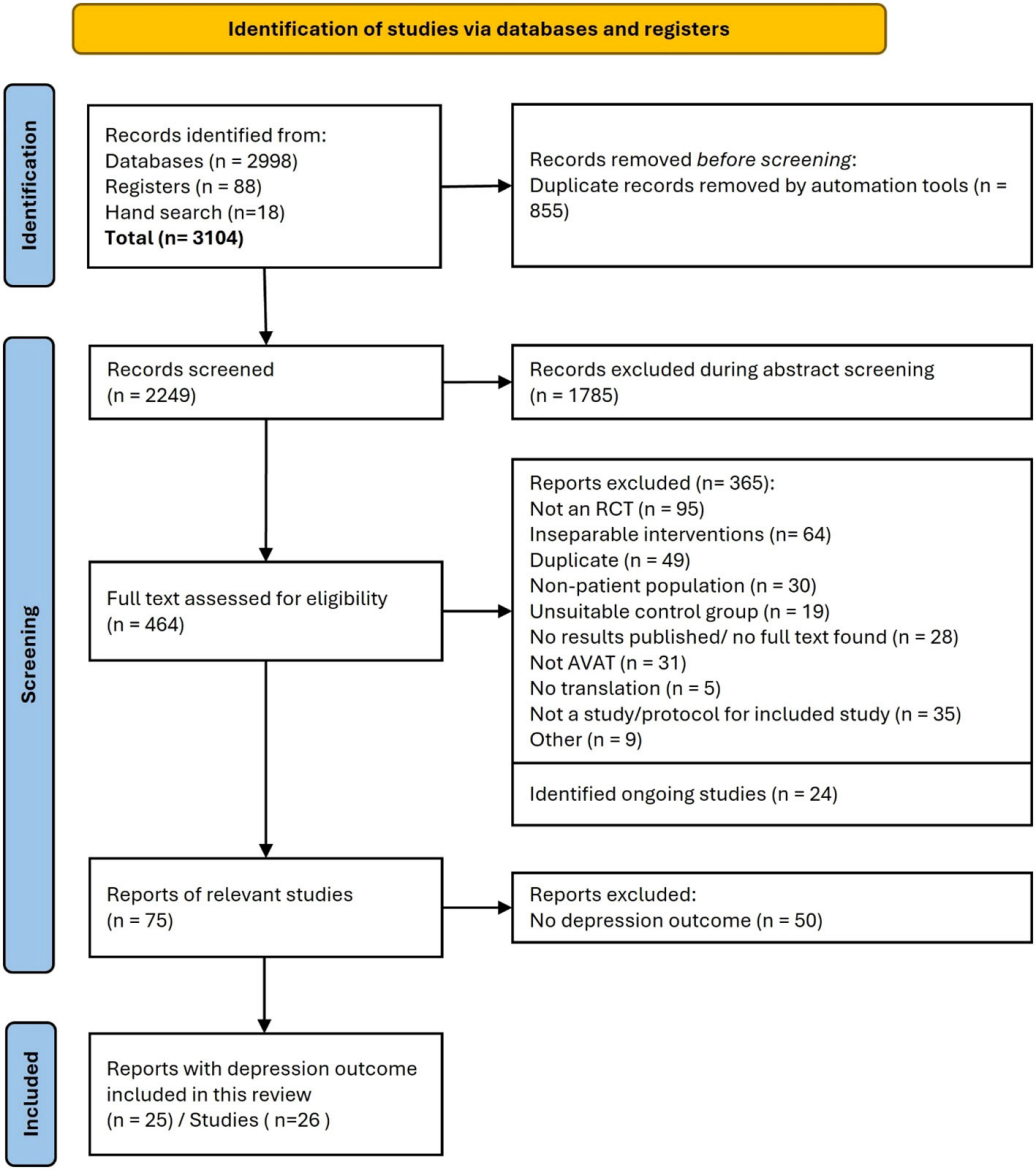
PRISMA flow chart of included studies.

We identified 3,104 records during our initial search, of which we excluded 855 as duplicates using Covidence (29) and Endnote (30). Two researchers independently screened 2,249 titles and abstracts, and subsequently 464 full text manuscripts. 365 records were excluded. Another 50 records were excluded for the purpose of this analysis, as they did not measure depressive symptoms as an outcome. We identified 25 reports of 26 studies for inclusion into our analysis. One paper (31) reported two different studies (**Figure 1**).

### 3.2 Included studies characteristics

The characteristics of the identified studies can be seen in **Table 1**. In only eight of the 26 studies, patients received treatment primarily because they suffered from depression or unspecified “mental illness”, including depression. Depressive symptoms were measured using Beck Depression Inventories (BDI-II and BDI-Y), Montgomery-Asberg depression rating scale (MADRS-S), Geriatric Depression Scale (GDS), Hospital Anxiety and Depression Scale (HADS)- depression subscale, Questionnaire of Depressive Conditions, depression-no depression subscale, Glasgow Depression Scale, Calgary depression scale for schizophrenia (CDSS) and for Dementia (CSDD), Behavior Assessment System for Children, Second Edition (BASC-2), Allgemeine Depressionsskala lang ADS-L, and Hamilton Depression Rating Scale (HAMD).

**Table 1:** Study characteristics of identified studies with depression as an outcome (TE, Therapeutic element, TAU, treatment as usual, Int, Intervention group, Con, Control group, AMT, Abbreviated Mental Test, BAI, Beck Anxiety Inventory, BDI, Beck Depression Inventory, GDS, Geriatric Depression Scale, HADS, Hospital Anxiety and Depression Scale, HARS, Hamilton Anxiety Rating Scale, HDRS, Hamilton Depression Rating Scale, MADRS, Montgomery-Åsberg depression rating scale, MMSE, Mini Mental State Examination, PTSD, posttraumatic stress disorder, SCL-90, Symptom Checklist 90 items, STAI, State Trait Anxiety Inventory).

Study	Population	Country	Number of Participants in analysis, sex distribution, group allocation	Age (Years) (Mean, range)	AVAT Intervention	Present core components of TE	Treatment duration	Control Group Activity	Outcome parameter	Follow-up
Beebe et al., 2010 (51)	Children with asthma, selected from their school campus, and their parents	USA	N=22 No further information reported	7-14	Art intervention with warm-up, art, and discussion phases. The children receive artificial jewels with symbolic meaning to increase their self-esteem. They do paper-mâché, clay, draw, paint, paint on masks to symbolize feelings, identify colours that make them feel good, use affirmative sentences, decorate craft objects, etc.	Patients share their feelings related to the art they created	7 1-hr weekly group sessions	Waiting list	The Beck Youth Inventories 2^nd^ Ed. (components disruptive behaviour, anger, depression, anxiety, self-concept)	6 months
Blomdahl et al., 2018 (52)	Adults with moderate to severe depression without psychotic symptoms	Sweden	N=79 23 male 56 female Int=43 Con=36	n in age groups 18–25, 9 26–35, 20 36–45, 20 46–55, 23 56–65, 7	Manual-based phenomenological Art Therapy additional to TAU	Manual-based, In-depth exploration of emotions and State of mind, Evaluation of treatment and process with focus on patient’s interpretation	10 1-hr weekly individual sessions	TAU (could include acupuncture, cognitive behavioral therapy, electroconvulsive therapy, interpersonal therapy, occupational therapy, pharmacological therapy, physical activity recipe, physiotherapy, psychodynamic therapy, and supportive therapy)	MADRS, Scale for Suicide Ideation (SSI)	no
Ching-Teng et al., 2019 (10)	Older adults with intact mental functions and mild depression	Taiwan	55 26 male 29 female Int=29 Con=26	78.4	Handiwork involving paper, clay, fingerprints, sandpaper, painting, collages, watercolor, and crayons	Sharing the meaning of creation and reflect on feelings of physical aging	12 1.5-hr weekly group sessions	TAU: Control group received the daily care of the long-term care institutes	Short GDS	no
Choi et al., 2020 (53)	Adults receiving pharmacotherapy for major depressive disorder	South Korea	35 7 male 28 female Int=19 Cont=16	Int=32.4 Con=36.6	Drawing (art psychotherapy) & TAU (pharmacotherapy)	Identification of emotions and free-associated feelings, reflection of interpersonal issues	6 1-hr weekly sessions (unclear whether group or individual setting)	TAU (pharmacotherapy)	BDI-II HDRS HARS BAI	no
Ciasca et al., 2018 (39)	Elderly women with major depressive disorder	Brazil	56 Int=31 Con=25	Int=66.1 Con=69.8	TAU and various crafting techniques (painting, mandalas made of grains, paint through straws, integrating rocks into artworks, drawing, clay modelling, weaving, collage, etc.) were used	Verbal expression of reflections and any feelings surfacing during the creative process	20 1.5-hr weekly group sessions	No intervention/TAU; control group did not receive any type of psychotherapy but was stable on pharmacotherapy	GDS BDI BAI	no
Decker et al., 2019 (37)	US veterans with PTSD	USA	31 30 male 1 female or other Int=16 Con=15	Int=43 Con=46	Art therapy (drawing images, visual trauma narratives) & cognitive processing therapy & TAU. Same hours spent with therapist in art/control group	Protocol-based, creation of visual trauma narratives, review of artwork	8 1-hr individual sessions	Cognitive processing therapy & supportive psychotherapy & TAU (pharmacological therapy for PTSD and Depression)	BDI-II	no
Ellis-Hill et al., 2019 (54)	≤ 2 years poststroke, community-dwelling adults and older adults	UK	47 in analysis Int=25 Con=22 56 entire cohort 32 male 24 female	69.8 27-88 (entire cohort)	TAU & art therapy working with paints, drawing materials, clay, textiles, and mixed media	Reconnection with internal selve, exploration of own senses	10 2-hr group session over 14 weeks	TAU: Support through the early discharge multidisciplinary team for the first 2–6 weeks after discharge, consecutively support from general practitioner and stroke coordinator	HADS	no
Gussak 2006 (31)	Adult prisoners (medium to maximum security), most taking medication for mental illness	USA	29 29 male 0 female	21-59	Drawing, mandala creation, sculpting, creating an “outside-inside” box, a poetry-pass etc.	Creation of self-symbols by drawing, exploring individual identity, fostering group-oriented behaviour	2 group sessions a day at 2 days a week over 8 weeks	Prison everyday life: No intervention/TAU	BDI-II	no
Gussak 2009 (55)	Adult prisoners, a majority having an axis 1 diagnosis and taking medication. Results are reported in one paper, but it was two studies, one conducted in a female, and one in a male prison	USA	158 62 male 96 female	Male: Int=22-50 Con=24-51 Female: Int=25-51 Con=20-47	Arts & crafts; name embellishing, self-symbol sculpture from paper, paper bridge, drawing, group mandala, self-box, etc.	Creation of self-symbols by drawing, exploring individual identity, fostering group-oriented behaviour	15 weekly group sessions	Prison everyday life: No intervention/TAU	BDI-II	no
Hattori et al., 2011 (56)	Older adults who consulted the outpatient clinic and showing mild impairment of cognitive function (MMSE score ≥ 20)	Japan	39 18 male 21 female Int=20 Con=19	Int=75.3 Con=73.3	Coloring of abstract patterns with pastel crayons or water-based paint, color line drawings of familiar objects (flowers, children, fish), drawing memory-based pictures	May be, not clearly reported	12 1-hr weekly group sessions	Learning therapy using calculation, involving simple calculation (additions and multiplications of 1- or 2-figure numbers)	GDS Apathy Scale	no
Ilali et al., 2019 (57)	Older adults, with normal cognitive function (≥7 AMT) and depression (≤ 11 SGDS) not taking medication or being stable on antidepressants for at least 3 months	Iran	54 22 male 32 female Int=27 Con=27	70	TAU & Art: Drawing and painting based on specific events in their lives. Materials: Paper, drawing pencil, color pencils, and oil pastels	Creative expression of life review, reflection of participants perceptions	6 1-hr weekly group sessions	TAU: Included quranic sessions, exercises, and psychotherapy meetings	GDS	1 month
Kopytin & Lebedev 2013 (32)	Hospitalized war veterans with non-psychotic mental disorders	Russia	112 102 male 10 female Int=62 Con=50	25-53 Int=38.0 Con=35.0	Group interactive art therapy (including drawing and painting) & TAU (psychopharmacological treatment and physiotherapy)	Release of emotions and stimulation, expression and understanding of life situation	12 to 14 2.5-hr group sessions 3 times per week	Occupational therapy & TAU (psychopharmacological treatment and physiotherapy)	SCL-90, Questionnaire of Depressive Conditions, Integrative Anxiety Test	no
Lalingkar et al., 2020 (58)	School children with learning difficulty, MARS attention score > 50 and depression score > 13	India	40 22 male 18 female	15 12-16	Coloring mandalas, clay modelling	Not reported	0.5-hr group sessions 4 times per week over 4 weeks	TAU: “conventional treatment, which included academic assistance by their teachers and normal rehabilitation exercises which focused on the associated developmental coordination disorders or other developmental disorders”	Glasgow Depression Scale	no
Lock et al., 2018 (59)	Adolescents with anorexia nervosa who have obsessive–compulsive features and respond poorly to family-based treatment	USA	30 3 male 27 female Int=15 Con=15	14.5	Family based treatment & manualized art therapy (incl. arts activities such as drawing)	Manual-based, expression and identification of emotions, reflection of process and outcome	15 0.5-hr sessions over 9 months (unclear whether group or individual setting)	Family based treatment & cognitive remediation therapy	Kiddie Schedule for Affective Disorders and Schizophrenia –Present and Lifetime Version, BDI BAI	no
Masika et al., 2021 (60)	Older adults with mild cognitive impairment (MCI), no literacy, and at increased risk of progression to dementia	Tanzania	39 5 male 34 female Int=21 Con=18	72.7	Visual art therapy (including drawing and Zentangle).	Expression of life experiences into art, reflection of experiences in focus groups	12 group sessions twice a week over 6 weeks	Health education on topics such as HIV, malaria, and tuberculosis	GDS short form	no
McCaffrey 2007 (33)	Older adults with depression (self-diagnosed or diagnosed by healthcare provider) and being able to walk 7–10 miles	Japan	40 No further information reported	75	Art therapy including drawing of self and one’s goals	Review and discussion of art work	6 group sessions over 6 weeks	Garden walks through a 200-acre garden which included 20 acres Japanese gardens, including koi ponds, waterfalls, and many places to sit and reflect	GDS	no
Montag et al., 2014 (61)	Adult inpatients with schizophrenia	Germany	35 22 male 13 female Int=16 Con=19	Int=38.8 Con=39.6	TAU & Psychodynamic Group Art Therapy	Reflection of art work	12 1.5-hr group sessions twice a week	TAU	Calgary depression scale for schizophrenia	3 months
Pongan et al., 2017 (62)	Older adults with probable Alzheimer’s disease (minor cognitive disorder or mild major cognitive disorder stage)	France	59 20 male 39 female Int=28 Con=31	Int=80.2 Con=78.8	Painting sessions followed by exhibition	Not reported	12 2-hr weekly group sessions over 3 months	Choral singing followed by concert gathering	GDS, STAI-trait, STAI-state	4 moths
Ramirez et al., 2020 (34)	Adolescent boys of color, living in poverty	USA	156 156 male 0 female Int=80 Con=76	9^th^ school grade No further information reported	Arts and crafts (collage work, self-portrait, landscape drawings, clay sculpting etc.), (method in detail reported in (63))	Expression of feelings regarding self and environment	12 weekly group sessions	Academic work assigned by teachers or interacting with each other	Behavior Assessment System for Children, 2^nd^ Ed. Self-Report of Personality	no
Roswiyani et al., 2020 (64)	Older adults living in nursing homes for at least 3 months, with a MMSE ≥ 18	Indonesia	267 84 male 183 female Int art alone=63 Int art+qigon=72 Con=132	73.8 50-96	Art activities (drawing, coloring, picture completion, mandala, collage), expression of feelings	Exploration of selve, expression of feelings, discussion of art work	16 1.5-hr group sessions twice a week over 8 weeks	Group 1 qigong, group 2 no intervention, just 90 min observation of patients	BDI-II GDS-15 items	no
Rusted et al., 2006 (35)	Older adults with dementia	UK	21 Int=9 Con=12	Men=80.3 67-92 Women =84.1 74-92	Group-interactive, psychodynamic art therapy approach that uses various art materials	Not reported	1-hr group session weekly over 40 weeks	Activity groups without “any formal occupational therapeutic methods or any form of art and craft work”	Cornell Scale for Depression in Dementia, Bond-Lader Mood Scale	3 months
Sterz et al., 2013 (65)	Inpatient rehabilitation patients with multiple sclerosis	Germany	69 21 male 48 female Int=37 Con=32	Int=37.3 Con=36.9	Arts and crafts (colors, gypsum, soapstone, clay)	Exploring inner psychological processes, exploration and stimulation of senses	1-hr group session twice a week over 3 to 10 weeks	Listening to relaxing music on CD on their own terms	Allgemeine Depressionsskala lang” ADS-L	6 months
Thyme et al., 2007 (40)	Adult women with depression or dysthymic disorder, not using psychopharmacological treatment	Sweden	39 0 male 39 female Int=18 Con=21	33.8 19-53	Art psychotherapy (psychodynamic focus)	Expression of feelings through art work, reflective dialogue between participant and therapist	10 1-hr individual sessions over 15 weeks	Verbal psychotherapy (psychodynamic)	SCL-90, BDI, HDRS	3 months
Tibbetts & Stone 1990 (36)	“Seriously disturbed” adolescents	USA	16 Int=8 Con=8	Int=14.6 Con=14.5	Art therapy according to the gestalt therapy principles	Raising awareness of blocked feelings and experiences	45-min weekly individual sessions over 6 weeks	Socialization sessions: Playing board games, talking about weekend activities, and taking walks around the school grounds	Burks’ Behavior Rating Scales, Roberts apperception Test for Children	no
Wu & Lee 2020 (38)	Adults diagnosed with PTSD	South Korea	93 57 male 36 female Int=50 Con=43	Int=22.0 Con=22.0	Conventional pharmacotherapy (TAU) & visual art therapy (sand play therapy and painting therapy, including therapeutic talking elements)	Expression of emotions, thought organization	1 individual session	Conventional pharmacotherapy (TAU)	HDRS HARS	3 months

The interventions ranged from individual manual-based art therapy in combination with pharmacological treatment to arts and craft activity groups with no psychotherapeutic elements. The control groups were equally heterogeneous and included verbal psychotherapy, pharmacotherapy, other treatments as part of the “treatment as usual” (TAU), waitlist, no intervention and other activities. Most studies were conducted in the USA (eight), followed by Sweden, the UK, Japan, and Germany (two each). One study was conducted in Taiwan, Brazil, Iran, Russia, India, Tanzania, France and Indonesia.

### 3.3 Meta-analysis

Of those 26 studies, 19 were suitable for quantitative data analysis. Reasons for exclusion from the meta-analysis were: Missing information about the interpretation of the measurement scale (32), qualitative results (33), a total dropout of more than 50% (34, 35), missing standard deviations (SDs) (36), and missing means and SDs (37–40) each reported two depression outcomes, of which the more population specific one was selected for the analysis.

### 3.4 Primary outcome overall effect

**Figure 2:**
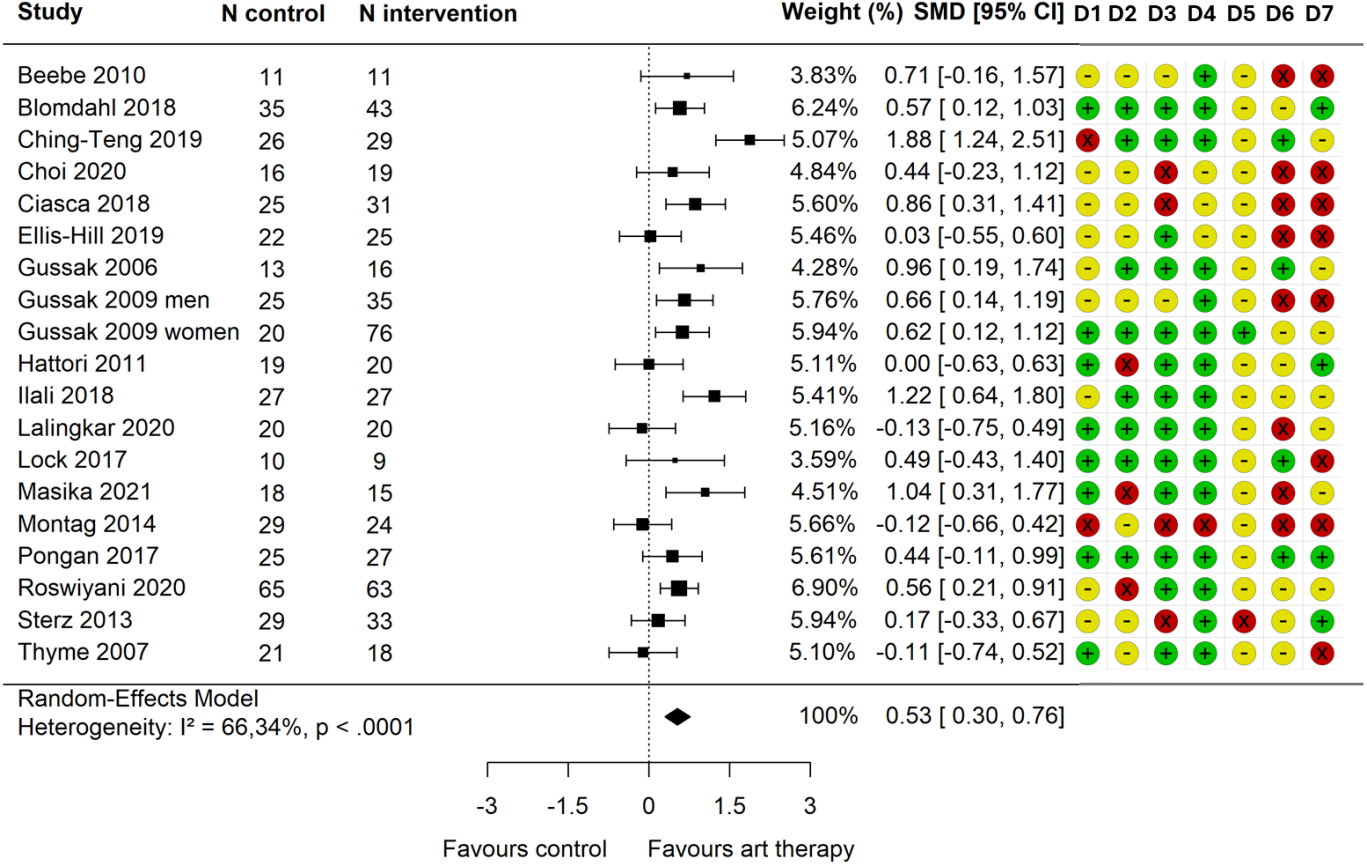
The effect of AVAT on depression, including bias. D1, Bias arising from the randomization process, D2, Bias due to deviations from intended interventions, D3, Bias due to missing outcome data or dropout, D4, Bias arising from the measurement tool, D5, Bias in measurement of the outcome, D6, Bias in selection of the reported result, D7, Other possible causes for bias. Colours represent: green=low risk of bias, yellow=unclear risk of bias, red=high risk of bias.

Even though a combined analysis of PT and CfB data using a pooled standardized mean difference (SMD) is usually not recommended, an analysis of 21 meta-analyses found that pooling PT and CfB data did not affect the results (41). Hence, we combined both analysis types to yield a more conclusive overview using R (42) and the packages metafor (43) and meta (44). The combined analysis of 997 patients indicated a medium effect for AVAT (SMD, 0.53, 95% confidence interval (CI): 0.30 to 0.76) (**Figure 2**). The statistical heterogeneity was statistically significant (*I²*, 66%, *p* < 0.001).

**Figure 3:**
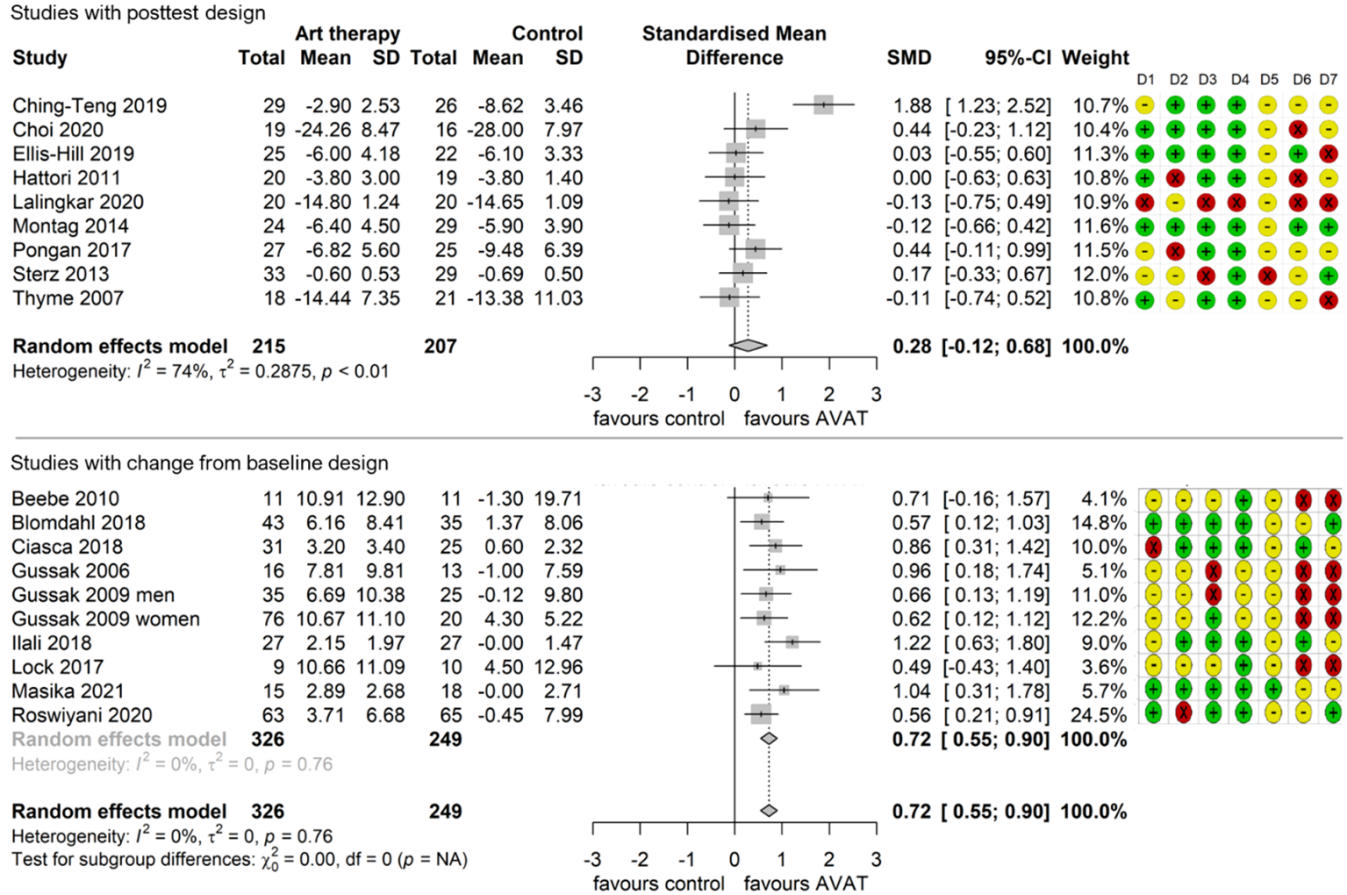
The effect of AVAT on depression, separated by study type (PT pictured on top, CfB below), including the domains of bias. D1, Bias arising from the randomization process, D2, Bias due to deviations from intended interventions, D3, Bias due to missing outcome data or dropout, D4, Bias arising from the measurement tool, D5, Bias in measurement of the outcome, D6, Bias in selection of the reported result, D7, Other possible causes for bias. Colours represent: green=low risk of bias, yellow=unclear risk of bias, red=high risk of bias.

A separate analysis of PT and CfB studies including associated risk of bias was performed (**Figure 3**). PT studies included 422 patients and displayed what would conventionally be considered a small overall effect size (SMD, 0.28, 95% CI: -0.12 to 0.68). However, since these are secondary explorative analyses, no statistical significance can be attributed to the results. CfB studies included 575 patients and indicated a medium effect (SMD, 0.72, 95% CI: 0.55 to 0.90).

### 3.5 Risk of bias

All studies included in the meta-analysis had at least one domain with high or unclear risk of bias (Cochrane risk-of-bias tool 2 for randomized trials (ROB2), **Supplementary Figure S1**). Visual inspection of the funnel plot and Egger’s regression test did not indicate evidence of publication bias (Egger intercept, 0.82, p, 0.64). (**Supplementary Figure S12**).

### 3.6 Secondary analyses

#### 3.6.1 Control groups

**Figure 4:**
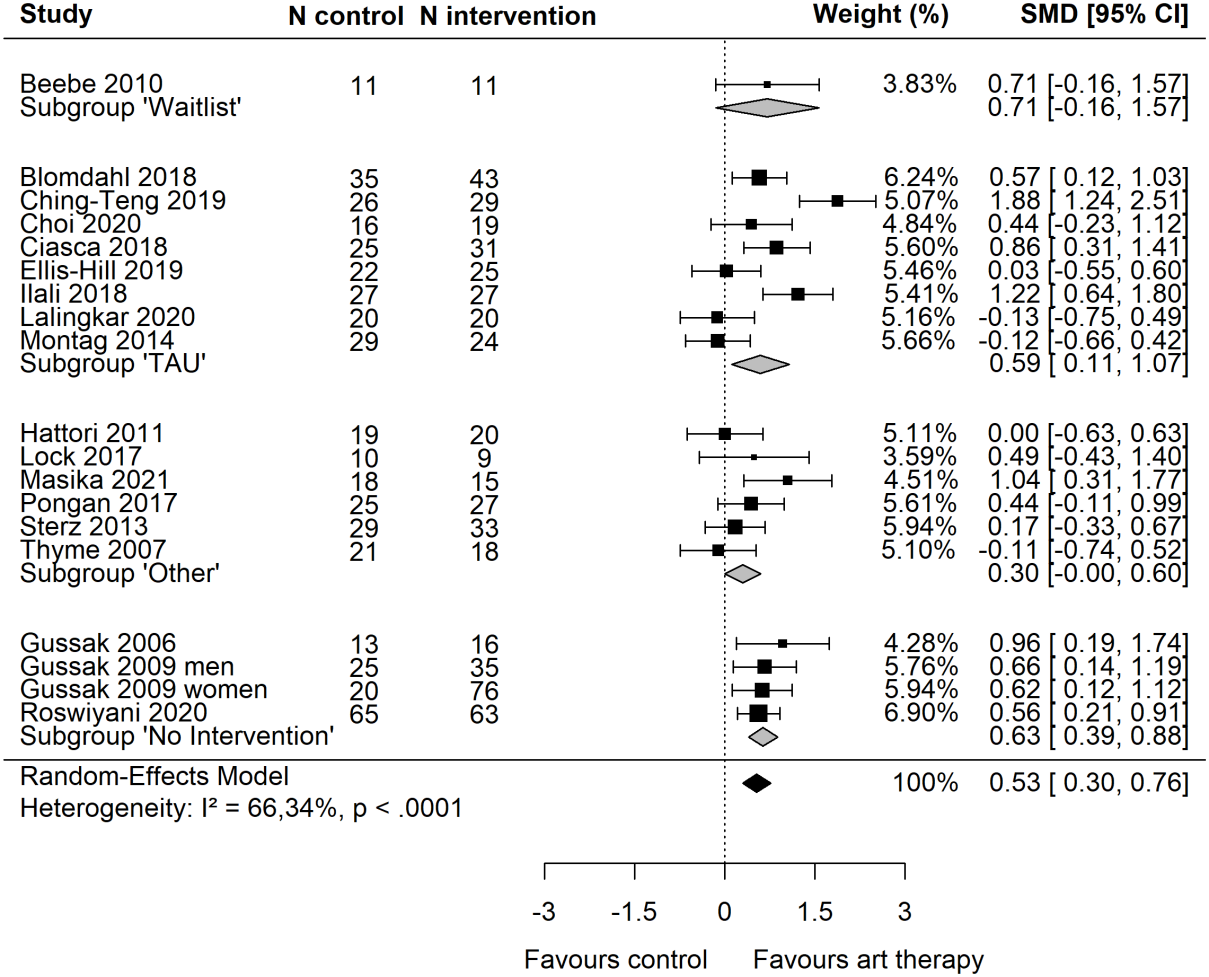
Subgroup analysis by control group.

The studies included in the meta-analysis used different types of control groups: “treatment as usual (TAU)” (eight), “no intervention” (four), “other” (six), and “waitlist” (one). Studies comparing AVAT to no intervention showed the largest improvement of depressive symptoms (SMD, 0.63, 95% CI: 0.39 to 0.88), followed by studies comparing to TAU control groups (SMD, 0.59, 95% CI: 0.11 to 1.07). Comparators consisting of “Other” types of controls (such as attention control, or other interventions) had a SMD of 0.30 (95% CI: 0.00 to 0.60). There was only one study with a waitlist control (**Figure 4**, **Supplementary Figures S2**, **S3**).

#### 3.6.2 Relevance of therapeutical element

**Figure 5:**
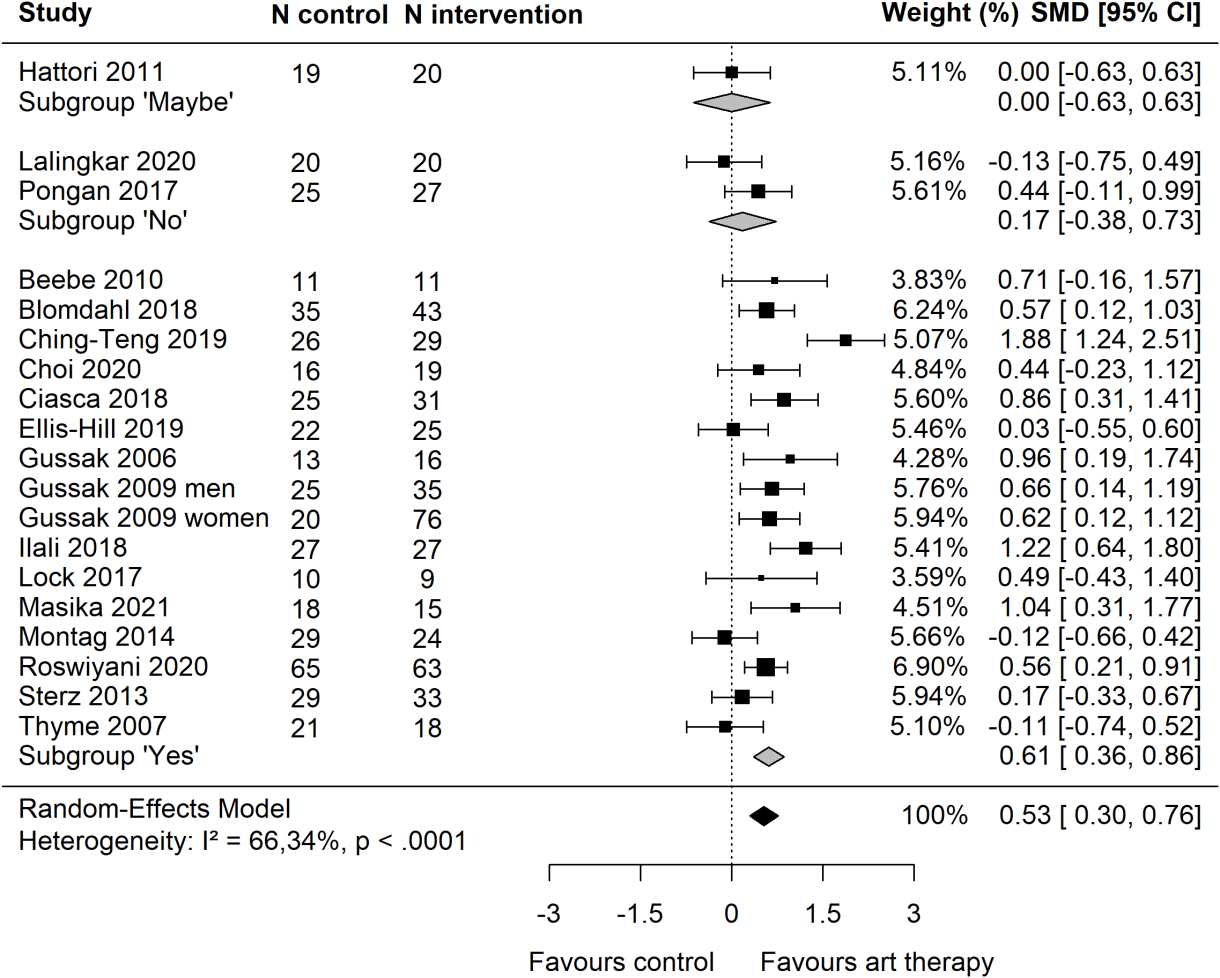
Subgroup analysis by use of intervention with a therapeutical element (TE).

Studies in which the intervention included a therapeutical element (TE) showed more improvement in the outcome, when compared to the control groups (SMD, 0.61, 95% CI: 0.36 to 0.86) than the study with uncertain TE usage, and studies without a TE (SMD, 0.17, 95% CI: -0.38 to 0.73) (**Figure 5**, **Supplementary Figures S4**, **S5**).

#### 3.6.3 Relevance of treatment setting

There were mixed results regarding the potential impact the treatment setting had on the intervention. Studies conducted in prison, assisted living facilities, educational settings, and not specified settings, tended to show positive associations, two studies conducted in inpatient settings showed no change, whereas studies conducted with outpatients showed great heterogeneity with an overall improvement of symptoms (**Supplementary Figure S6**).

#### 3.6.4 Relevance of intervention duration

Sixteen studies reported enough data to calculate the total intervention duration in minutes. We calculated a random effects meta regression to assess the relationship between total time spent in the intervention and effect size. The total intervention duration varied widely across studies, with the shortest intervention lasting for a total of ca. 6 hours (~ 360 minutes), and the longest accumulating to ca. 30 hours (~1800 minutes) (**Supplementary Figure S6**). Intervention duration did not explain any of the variance between studies (*R²* < 0.001). The estimation coefficient for 10 intervention hours was 0.11 (standard error (SE), 0.19, 95% CI: −0.26 to 0.48) and not associated with effect size (*p*, 0.55)).

#### 3.6.5 Intention to treat and per protocol analyses

Overall, studies with PP (n=14) showed a slightly stronger improvement of depressive symptoms with the use of AVAT (SMD, 0.55, 95% CI: 0.25 to 0.85) compared to studies with ITT (n=5) (SMD, 0.41, 95% CI: 0.11 to 0.73) (**Supplementary Figure S7**).

#### 3.6.6 Group setting

In most studies (n=15), AVAT was conducted in a group setting, two had individual sessions, and two studies did not report the setting. Group therapy might be associated with a slightly increased treatment benefit when compared to studies with an individual setting or studies that did not specify the number of people (**Supplementary Figure S8**).

#### 3.6.7 Age

Thirteen studies reported a mean age of their participants, which averaged 55.4 years. Overall, older adults and adults profited slightly more from the treatment than children/adolescents (**Supplementary Figure S9**).

#### 3.6.8 Further subgroup analyses

Subgroup analysis according to treatment indication and assessment scale yielded mixed results (**Supplementary Figure S10**). The studies included six different clinician assessed scales and four different self-reported scales as diagnostic instruments. Clinician assessed scales tended to slightly favor AVAT over self-reported scales (**Supplementary Figure S11**).

We conducted a subgroup analysis of studies if their patient population had depression as the primary treatment indication. Gussak 2006 and Gussak 2009 did not specify the exact treatment indication but described most of the population as “taking medication for mental illness” (17) and “the majority of the inmates have received Axis I diagnoses” (25) and were therefore included. A total of nine studies met these criteria. The improvement in symptoms for this subgroup was larger (SMD, 0.78, 95% CI: 0.43 to 1.14) when compared to the total improvement of symptoms across all studies.

## 4 Discussion

Overall, AVAT was associated with an improvement in depressive symptoms in the studies included in this review. The medium effect size of art therapy shown by the pooled standardized mean difference (SMD) is comparable to that of other therapeutic approaches for mental health disorders, such as antidepressants for postnatal depression (45), exercise for depression (46), or psychological interventions for depression (47). However, given the high variance and bias in the results and the evidence is uncertain. Multiple factors could have influenced the effect of AVAT in the included studies, including analysis methods, outcome assessment, population and intervention characteristics, and therefore contributed to the heterogeneity in results.

### 4.1 Discussion of heterogeneity

In determining the effect of AVAT, the two most important components are the chosen AVAT intervention and control group, against which the AVAT is being compared. The interventions of the included studies were very diverse, ranging from coloring-in shapes to art psychotherapy. Similarly, the settings in which the AVAT took place, were ranging from prison to nursing homes, given by therapists with varying qualifications and backgrounds. The control groups also displayed great variation, from groups receiving no treatment, TAU groups including psychotherapy and pharmacotherapy, to controls engaging in other activities, such as choral singing.

Strong and potentially therapeutic control activities, such as psychotherapy and singing, may have diluted the true effects of AVAT, leading to a potential underestimation of effect in our dataset. Comparing AVAT to TAU or to no intervention might be the most appropriate type of control group when trying to reflect real world clinical settings. It is therefore remarkable that studies with TAU control groups showed greater improvements under AVAT, given they might be the most suitable comparators. This should inform the design of future intervention studies.

Perhaps not surprisingly, AVAT with a TE showed greater improvements than interventions without it. Art therapy traditionally integrates some TE into the art making. The patients talk about their artwork, their creative process, or reflect on emotions that become visible through the art. Some included studies were missing that crucial TE. Conversely, some of the identified control groups included such TE. This might have led to some beneficial effects for control groups with a TE and weakened the effect of intervention groups that were missing a TE.

The issues of a child with asthma are vastly different from the problems a prison inmate with a substance use disorder might face. These very heterogeneous patient groups, being treated for different indications in varying treatment settings will likely have contributed to the nonuniform results.

Interestingly, in our sample, outpatients profited more from the intervention than inpatients. In clinical praxis, access to AVAT is significantly more difficult for outpatients, whereas art therapy is commonly offered to patients who stay in hospitals (48). Our analysis suggests that there might be some benefit in facilitating access to art therapy for outpatients, since it might help reduce depressive symptoms, and its potential is currently not yet fully realized for this patient group.

The way in which the outcome was assessed might have played a role as well: Depressive symptoms assessed by a clinician tended to show a larger improvement than self-report measures, which is in line with existing literature (28). When following a patient centered approach, it might be more meaningful to focus on self-report measures to reflect the lived experience of the patients.

Studies with a CfB approach showed larger effect sizes than studies with PT measurements, probably because change scores reduce some of the inter-person variability (41). In addition to the methodological causes, the weaker effect of the PT studies might also be explained through additional factors: Studies without TE, or with potentially healing control activities were all PT. This could have contributed to an overall weaker PT result.

### 4.2 Comparison of the results with previous findings

The overall effect of 0.53 SMD calculated in this meta-analysis for AVAT in the treatment of depressive symptoms corresponds to a moderate effect size. Due to the wide variation in inclusion criteria and study objectives, a direct comparison with other reviews is not possible. The review by Barnish and Nelson-Horne is more cautious regarding the effect size. However, the authors did not conduct a meta-analysis because the heterogeneity of the included studies was very high. This is explained by the significantly broader inclusion criteria (creative art, performing art, music therapy, dance, etc.). Furthermore, non-randomised and observational studies were also included. Nevertheless, a potential benefit for AVAT was concluded (22). An older systematic review by Boehm and colleagues focused, among other things, on depressive symptoms in cancer patients (49). Here, too, all forms of artistic therapy were considered. With similarly high heterogeneity, an SMD of 0.3 was found, indicating a borderline moderate effect size on depressive symptoms.

Our subgroup analysis of studies with versus without an explicit therapeutic element demonstrated the importance of a therapeutic element in achieving superior efficacy compared with controls. This confirms the findings of a systematic review of various therapeutic elements in studies on depression (50). Although the authors did not quantify the effects, the relevance of the therapeutic elements was shown to be independent of the clinical setting in which AVAT was used.

### 4.3 Strength and limitations

This analysis has multiple limitations, primarily, being an explorative secondary analysis of the data. The quality of any meta-analysis depends on the studies included, and in this case, there was notable bias present, which may limit generalizability. Furthermore, sample sizes tended to be small, which could indicate potential publication bias.

The conclusions drawn from the subgroup analyses, which are based on a very small number of studies, are subject to considerable uncertainty; for example, there is only one study in the subgroup of waiting-list controls, and the subgroup of interventions without a therapeutic component contains only two studies. The subgroups covering different treatment settings are also mostly very small.

In contrast, the strengths of the review are the methodological thoroughness and wealth of data. We have conducted multiple subgroup analyses to investigate various variables of interest. By including only RCTs, we ensured greater comparability among the studies, and formally assessing study bias has further highlighted potential directions for future research.

## 5 Conclusion

Overall, there is suggestive evidence that art therapy as complementary treatment may reduce depressive symptoms. The inclusion of a TE in the intervention may increase the effectiveness of the intervention, while the total duration of the intervention might be of lesser importance. Patients who were specifically treated for depression had a greater reduction in depressive symptoms than patients with other primary treatment indications. Given the explorative nature of the analyses, the small study sample sizes, and high study level bias, treatment effects should best be determined in future large scale intervention studies.

## Data Availability

The original contributions presented in the study are included in the article/supplementary material, further inquiries can be directed to the corresponding author/s.
