# Supplementary material for: Art therapy for depression: a systematic review and meta-analysis
Source: Front Psychiatry. 2026 Jul 23;17:1789633. doi: 10.3389/fpsyt.2026.1789633 (PMC13443295; doi:10.3389/fpsyt.2026.1789633)
Supplement: Supplementary file 1 [file DataSheet1.pdf]

Supplementary Material

Art therapy for depression: A systematic review and meta-analysis

Ronja Joschko, Stephanie Roll, Weronika A. Grabowska, Anne Berghöfer, Stefan N. Willich

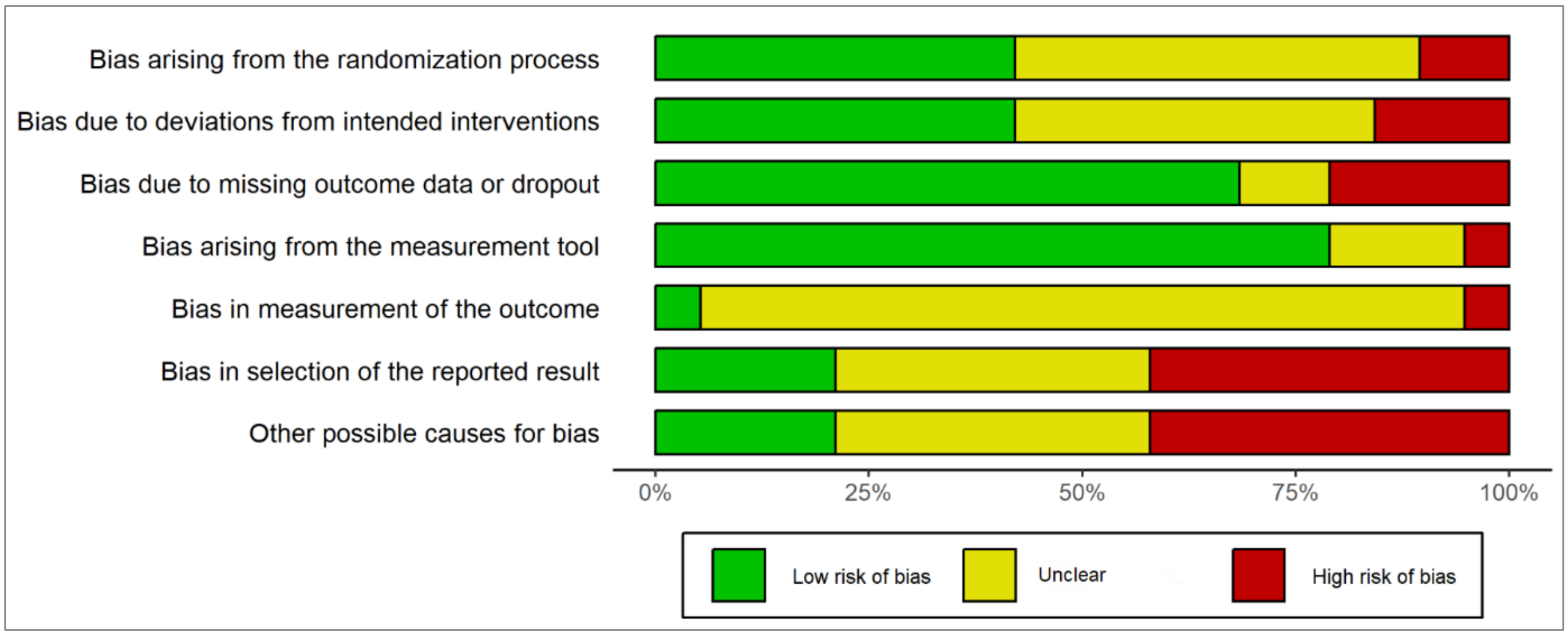

Supplementary figure s1: Customized risk of bias tool with seven domains of bias and overall risk of bias.

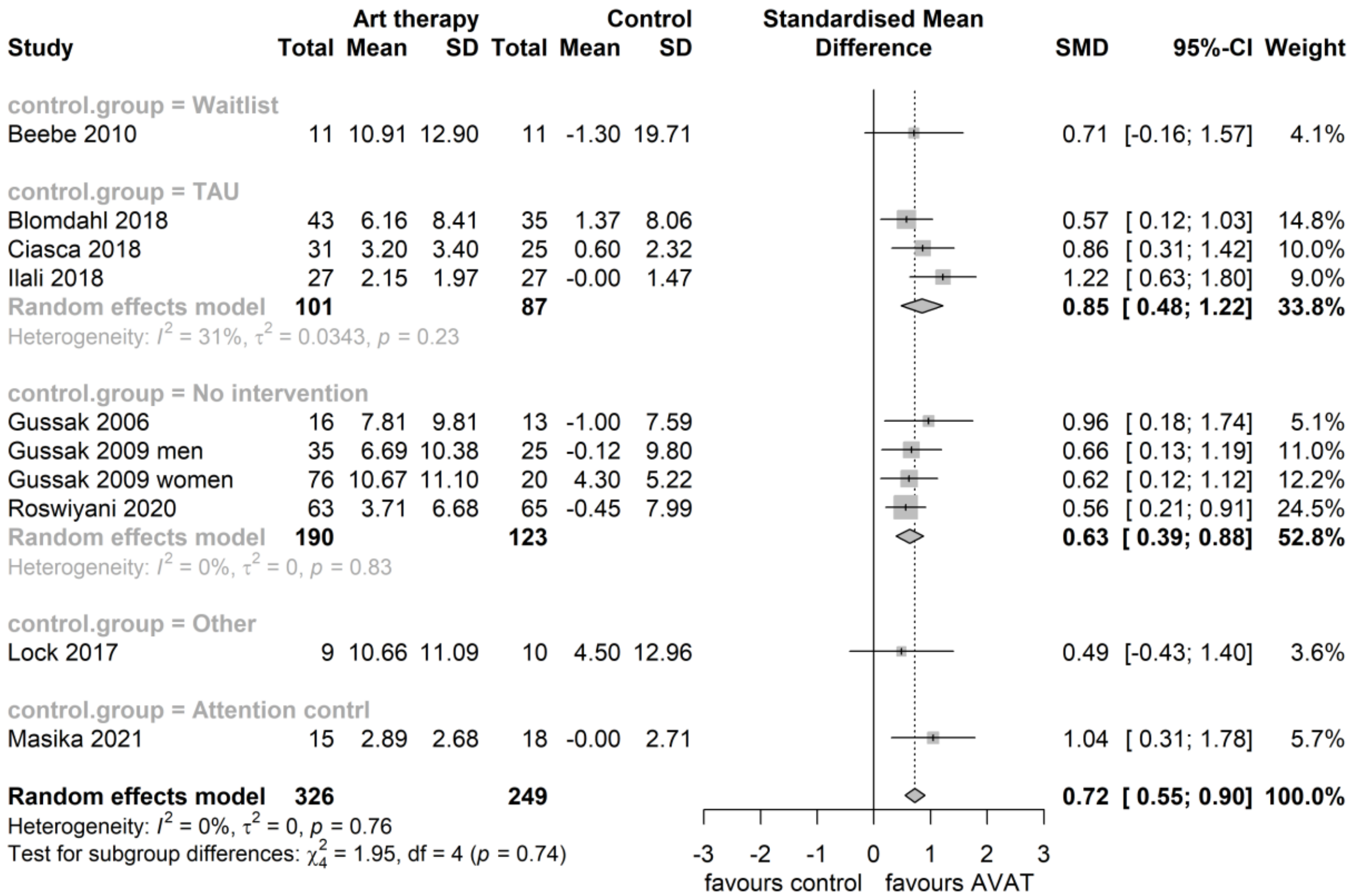

Supplementary figure s2: Subgroup analysis of CfB studies by control group type

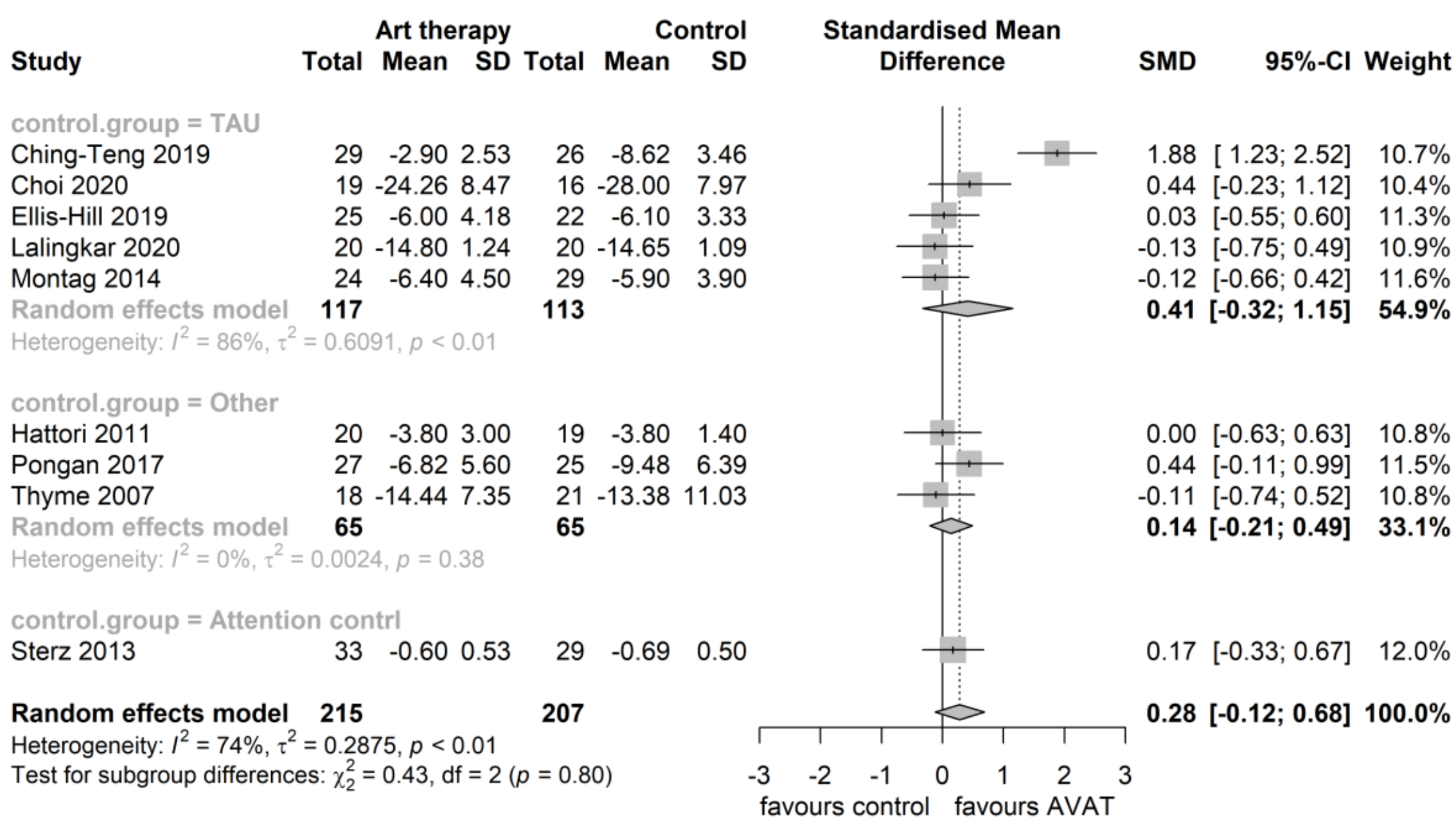

Supplementary figure s3: Subgroup analysis of PT studies by control group type

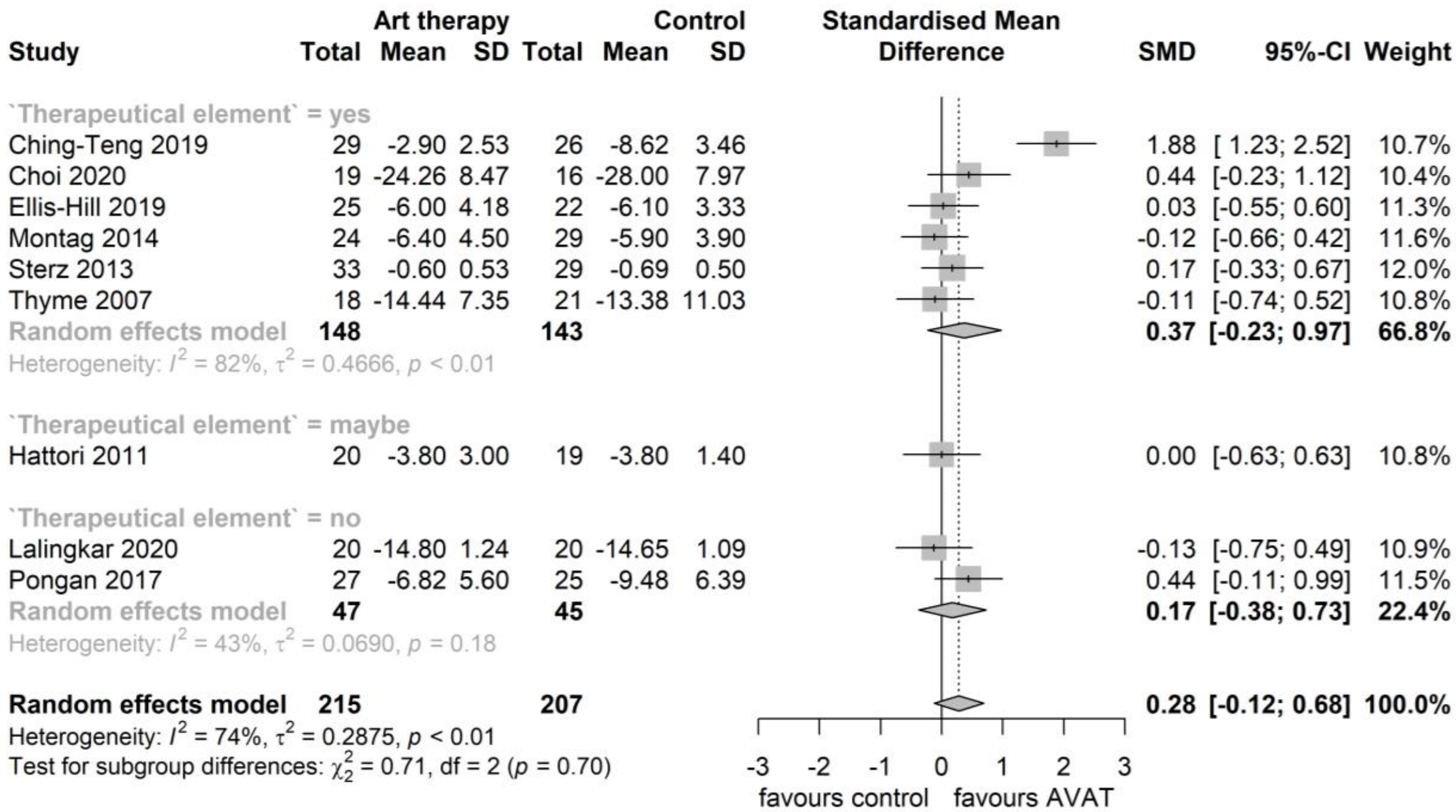

Supplementary figure s4: Subgroup analysis of PT studies according to the presence of a therapeutical element (TE) in the intervention.

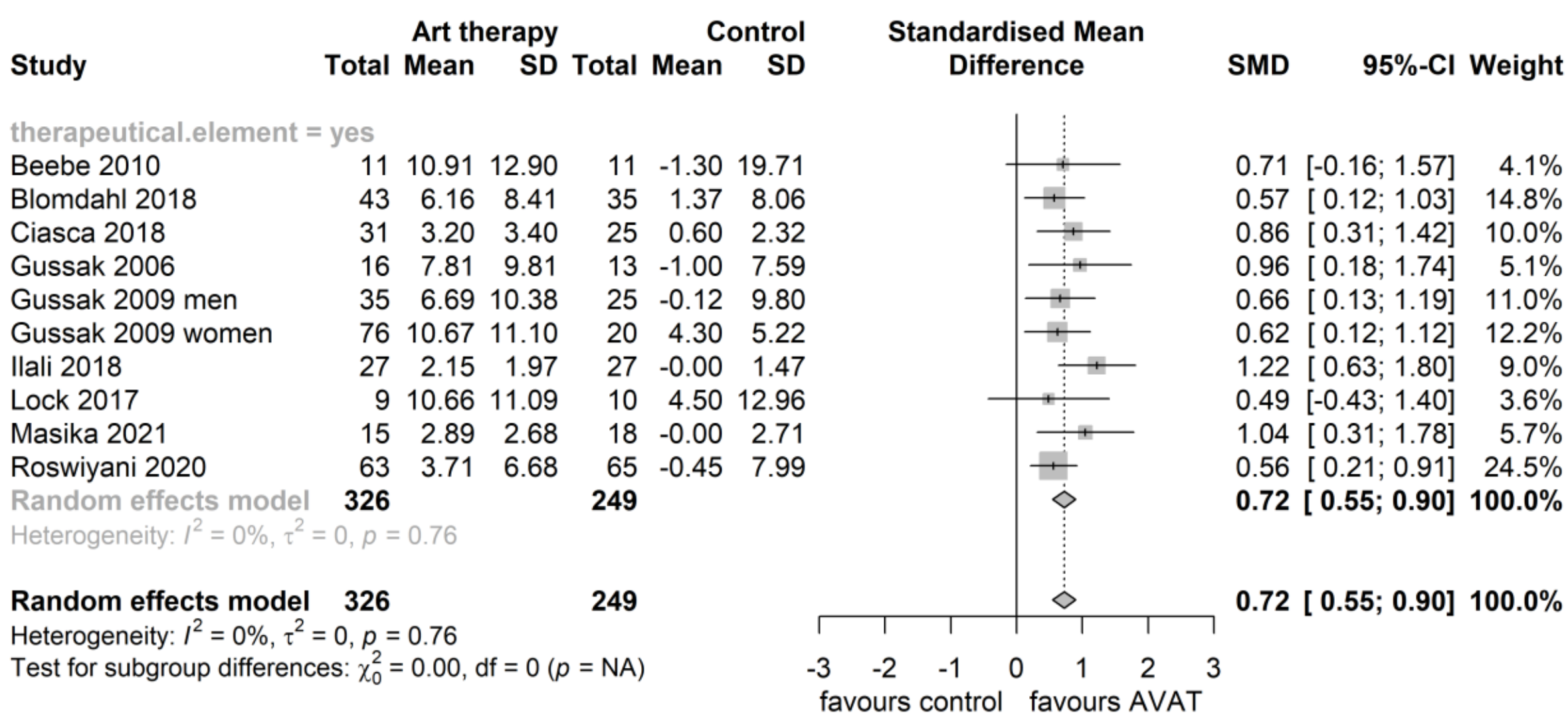

Supplementary figure s5: Subgroup analysis of CfB studies according to the presence of a TE in the intervention. All CfB studies contained a TE.

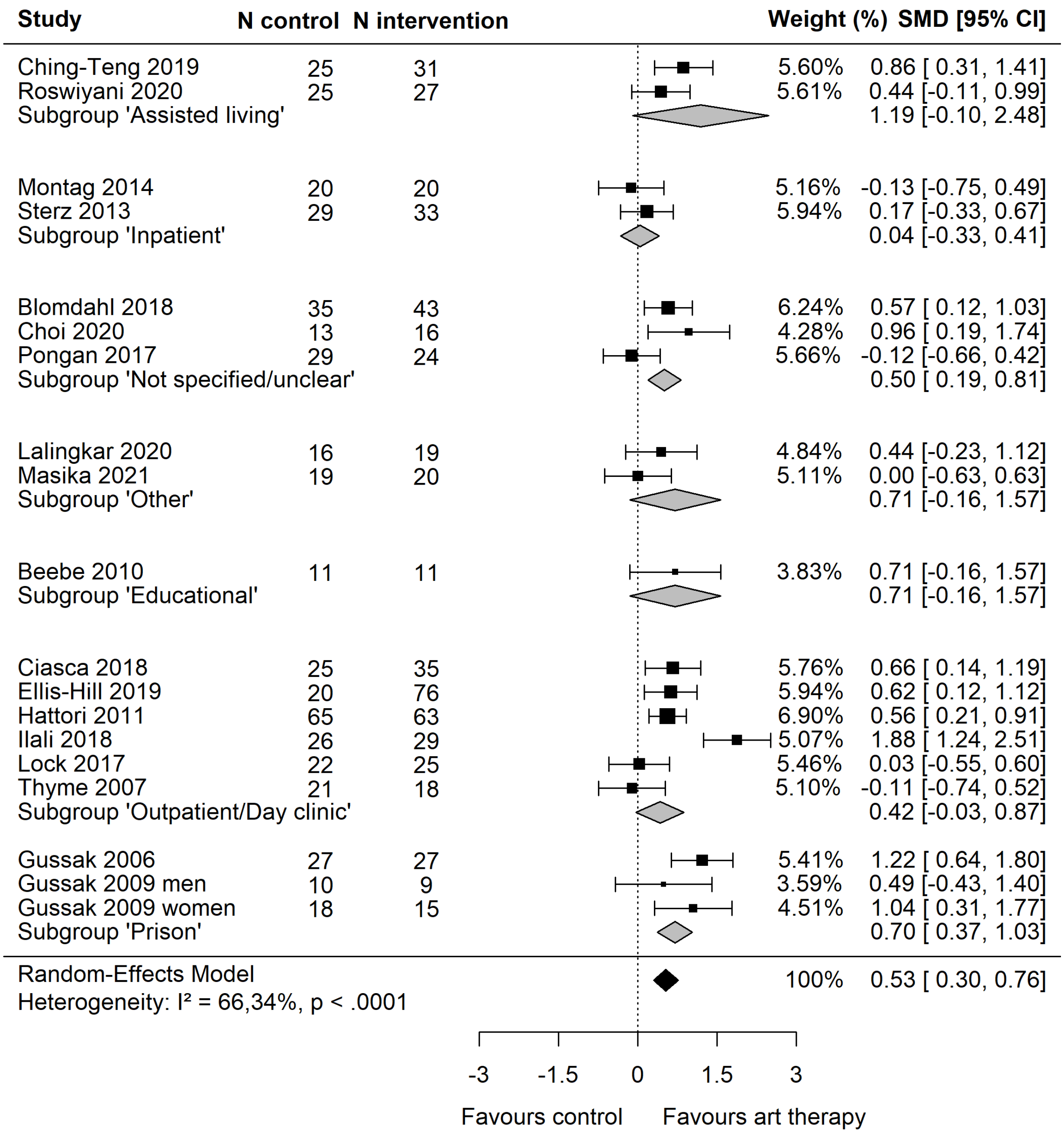

Supplementary figure s6: Subgroup analysis by treatment setting

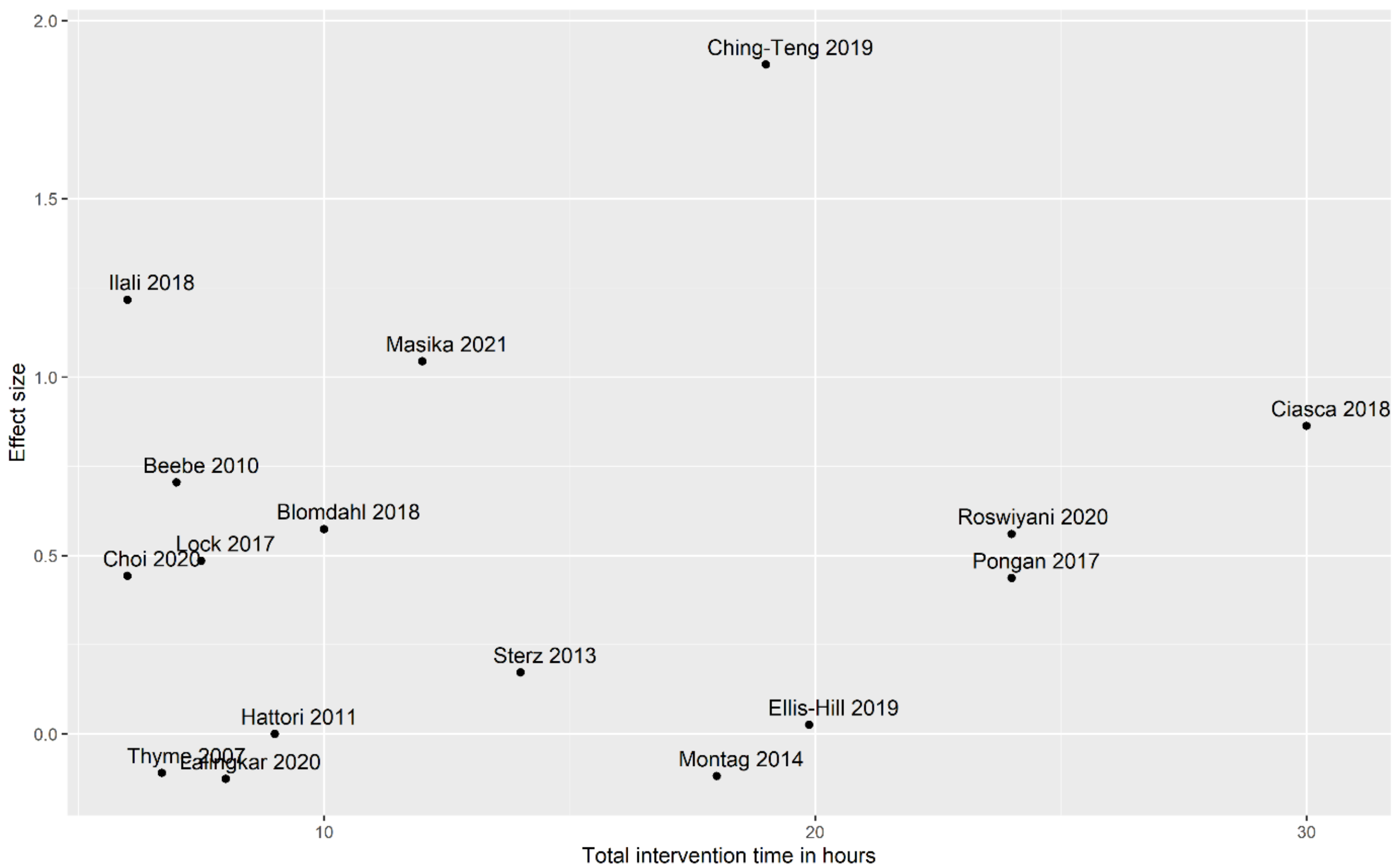

Supplementary figure s6: Total intervention duration in hours and effect size of the studies.

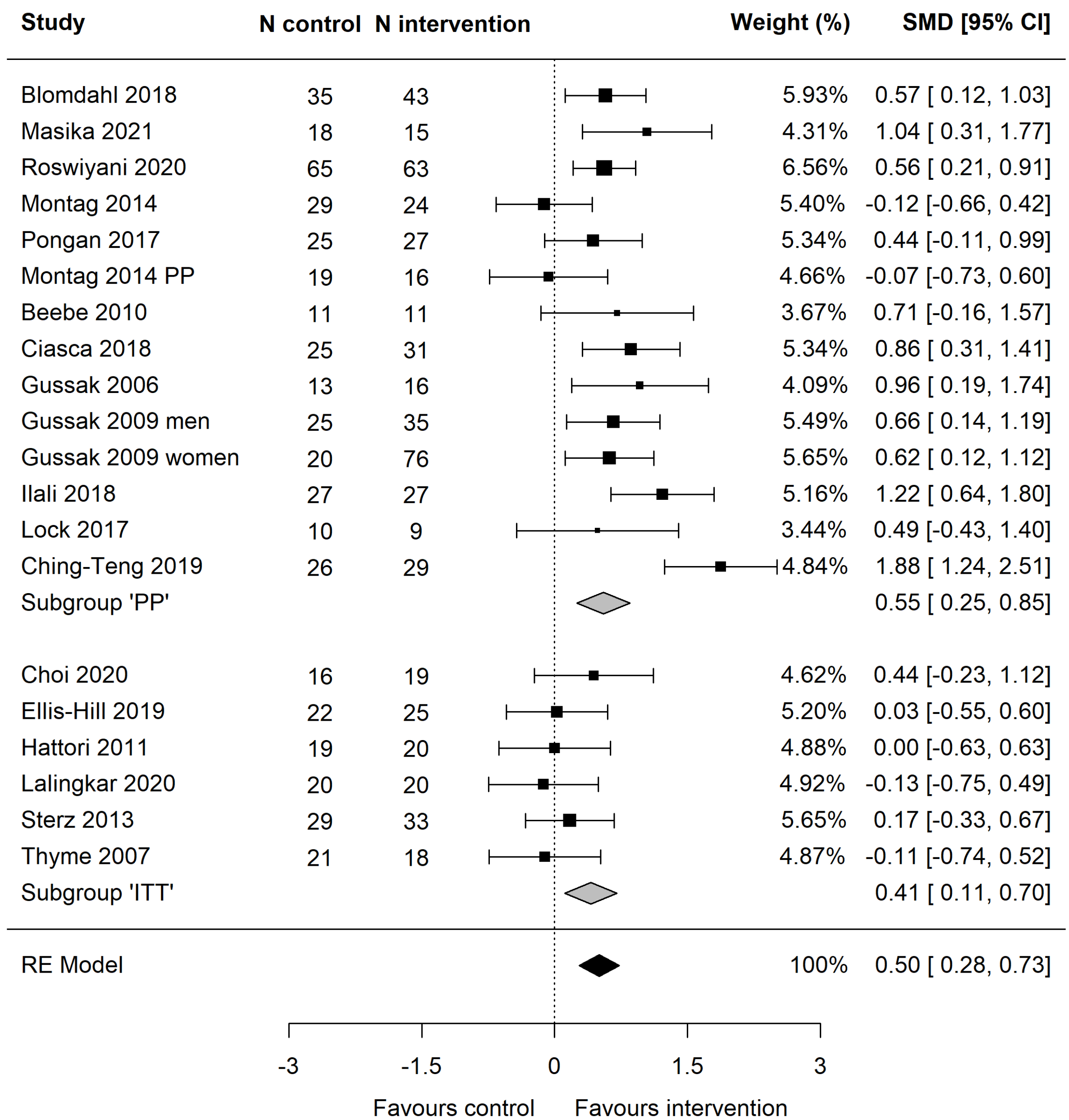

Supplementary figure s7: Combined effect for studies with ITT vs PP analysis.

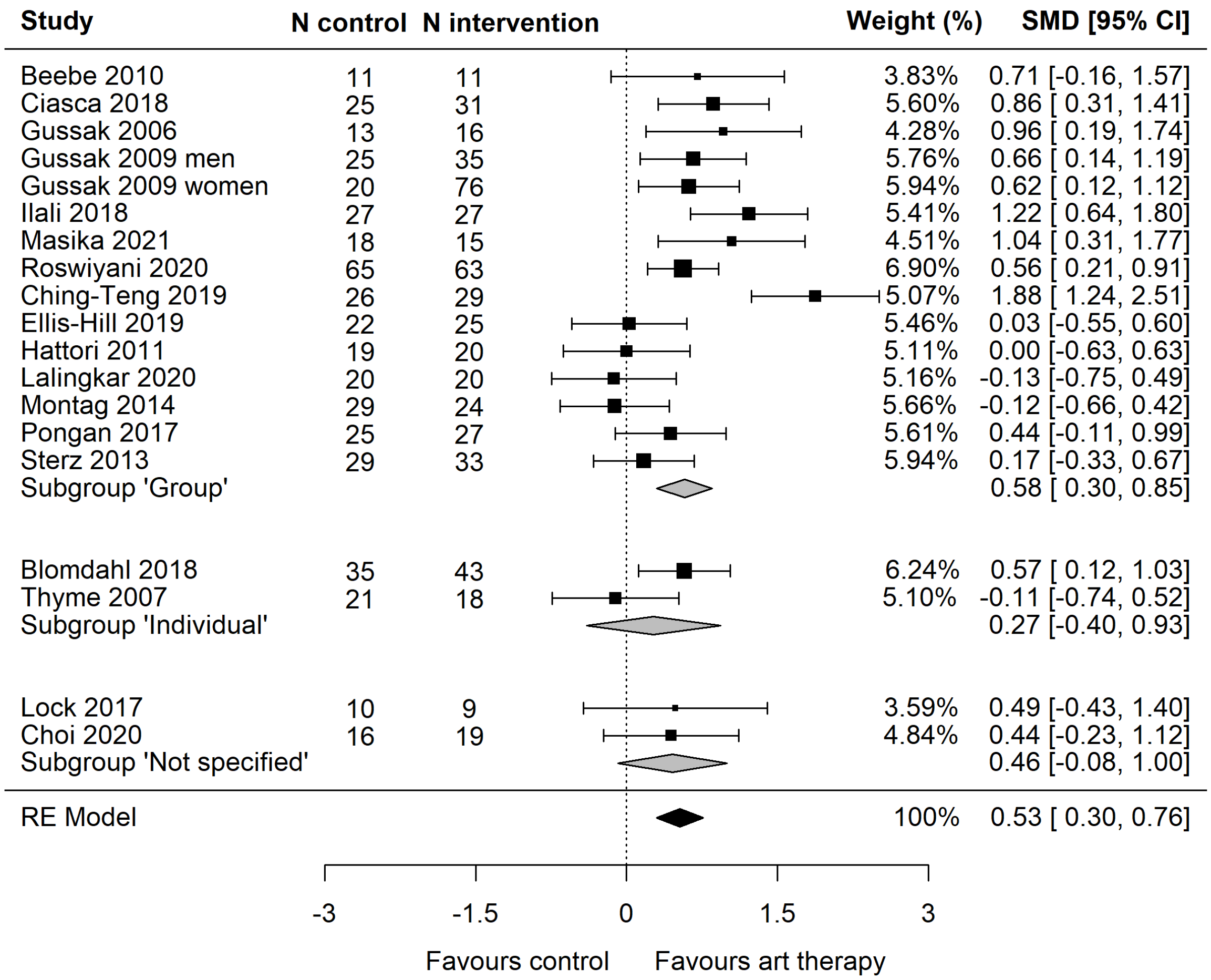

Supplementary figure s8: Combined forest plot of subgroup analysis of the group type used for the intervention.

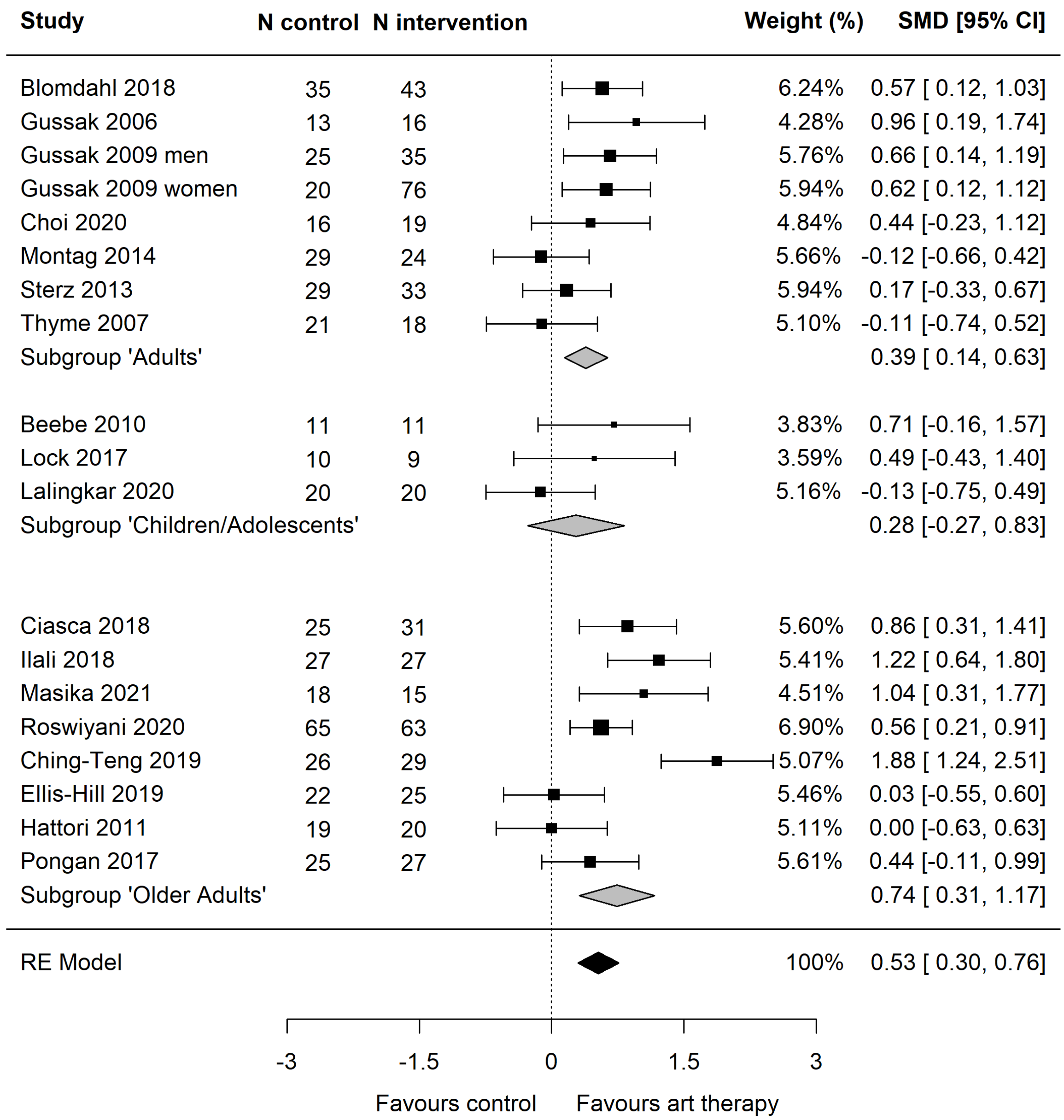

Supplementary figure s9: Depressive symptoms by age group

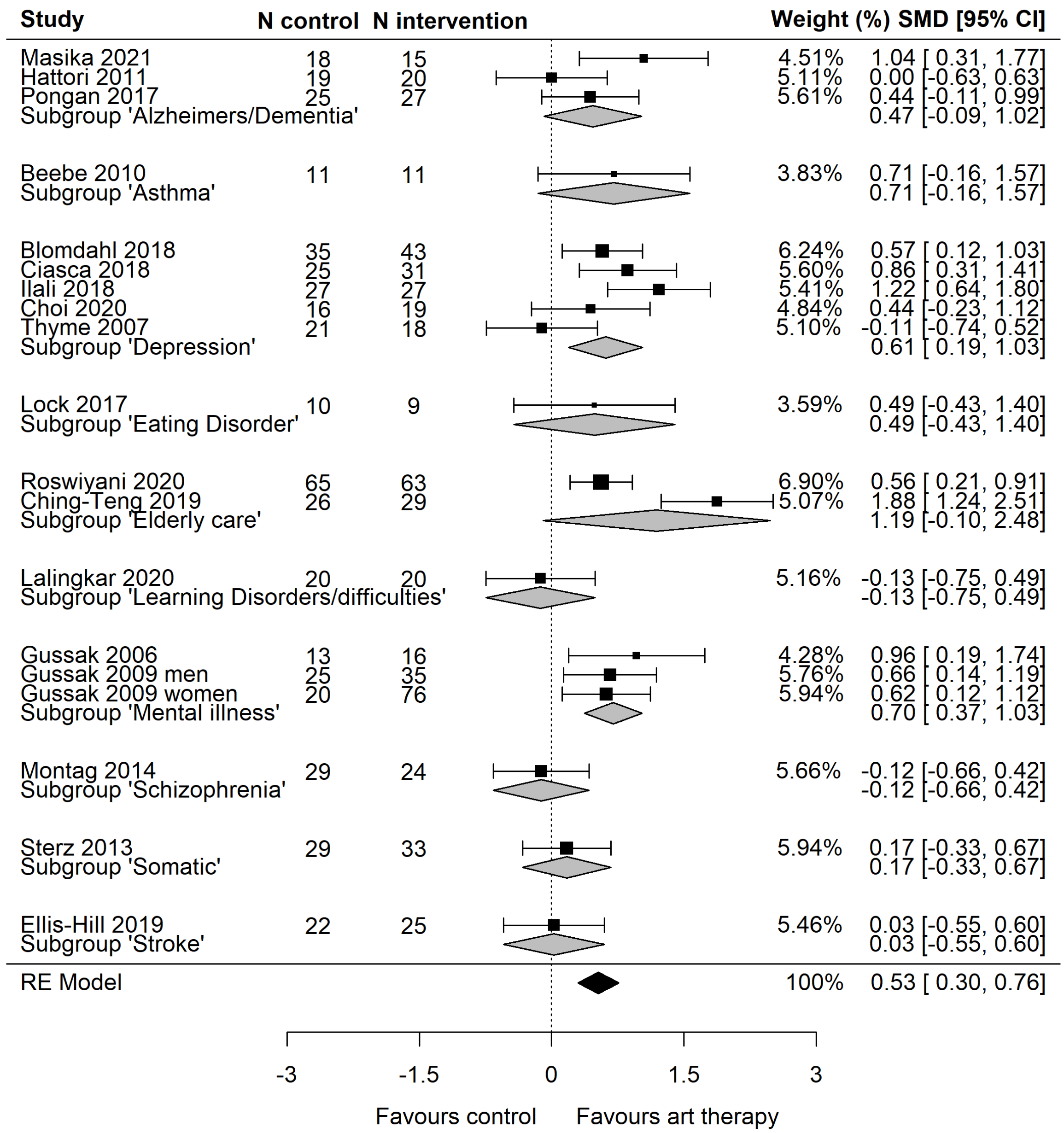

Supplementary figure s10: Subgroup analysis by treatment indication

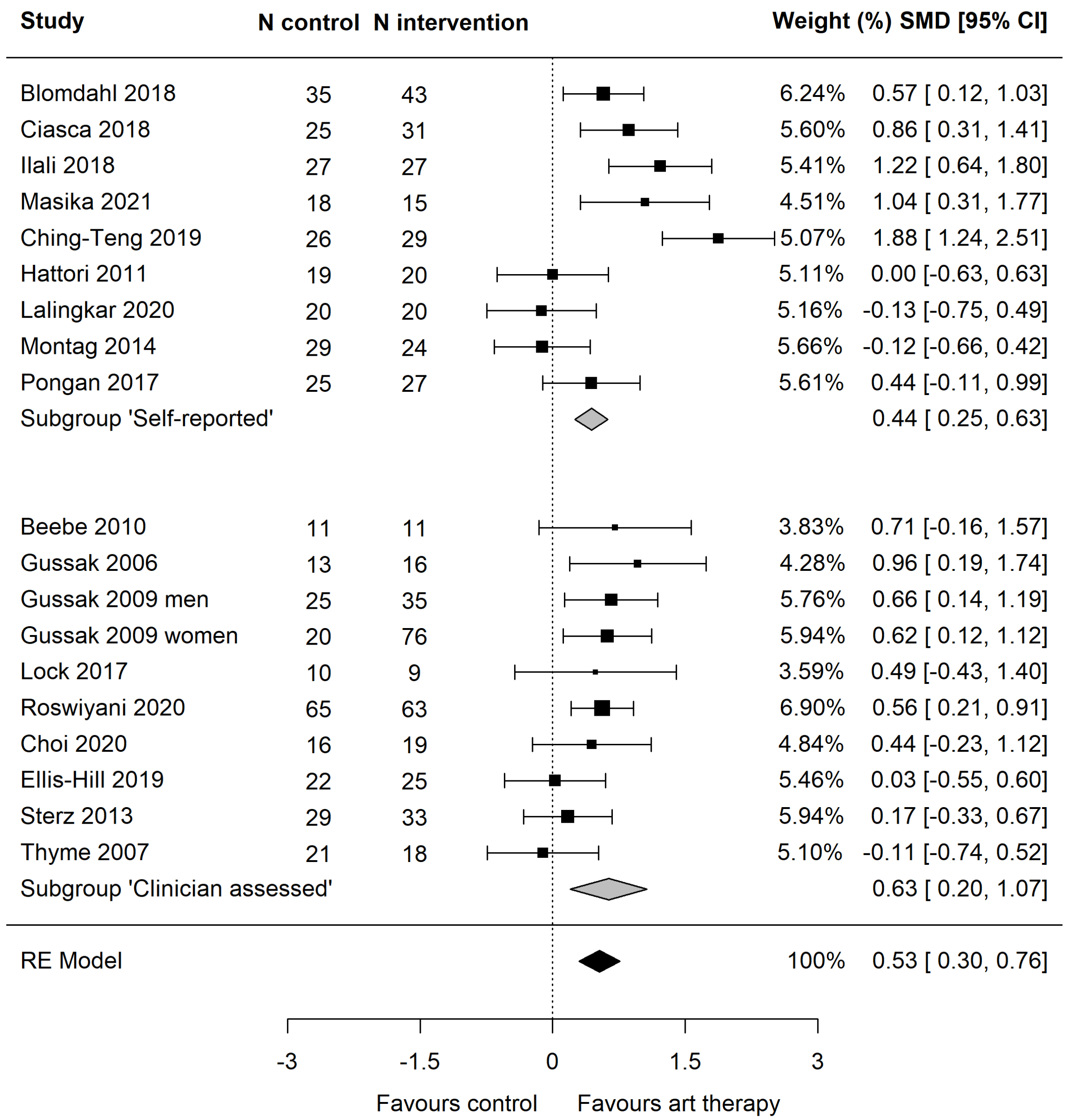

Supplementary figure s11: Studies using clinician assessed vs self-reported outcome assessment scales. Clinician assessed scales = Montgomery-Asberg Depression Rating Scale, Geriatric Depression Scale, Glasgow Depression Scale, Calgary Depression Scale for Schizophrenia, Cornell Scale for Depression in Dementia. Self-reported scales = Beck Inventory, Beck Inventory II, Hospital Anxiety and Depression, Allgemeine Depressions Skala

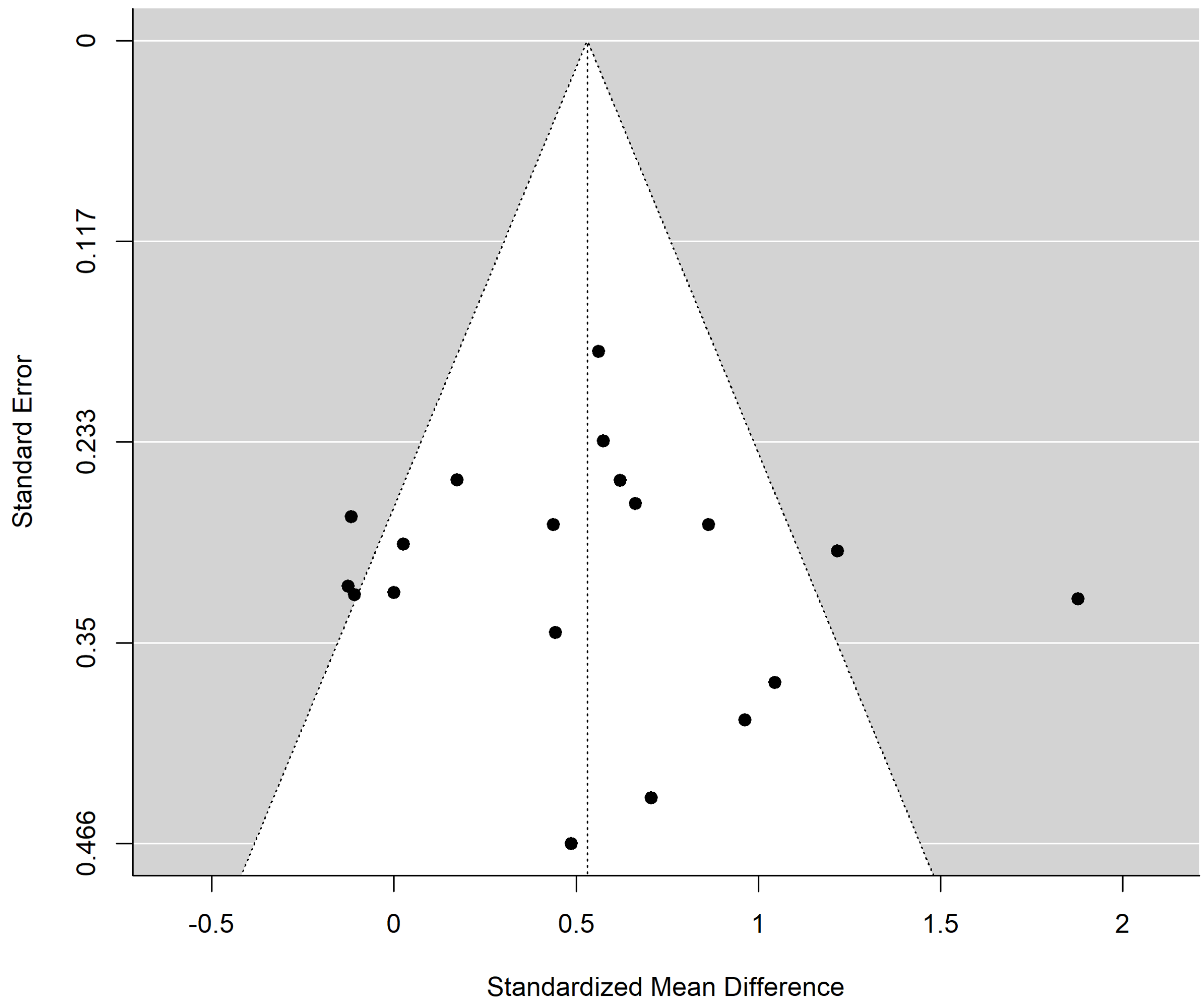

Supplementary figure s12: Funnel plot of published studies.
